# Fusarium Head Blight Modifies Fungal Endophytic Communities During Infection of Wheat Spikes

**DOI:** 10.1007/s00248-019-01426-3

**Published:** 2019-08-26

**Authors:** Edward C. Rojas, Rumakanta Sapkota, Birgit Jensen, Hans J. L. Jørgensen, Tina Henriksson, Lise Nistrup Jørgensen, Mogens Nicolaisen, David B. Collinge

**Affiliations:** 1 Section for Microbial Ecology and Biotechnology, Department of Plant and Environmental Sciences & Copenhagen Plant Science Centre, University of Copenhagen, Thorvaldsensvej 40, DK-1871 Frederiksberg C, Denmark; 2 Department of Agroecology, Faculty of Science and Technology, Aarhus University, Forsøgsvej 1, DK-4200 Slagelse, Denmark; 3 Section for Plant and Soil Science, Department of Plant and Environmental Sciences & Copenhagen Plant Science Centre, University of Copenhagen, Thorvaldsensvej 40, DK-1871 Frederiksberg C, Denmark; 4 Lantmännen Lantbruk, Onsjövägen 13, 268 31 Svalöv, Sweden

**Keywords:** *Fusarium graminearum*, FHB, Fungal communities, Endophytes, Metabarcoding

## Abstract

Fusarium head blight (FHB) is a devastating disease of wheat heads. It is caused by several species from the genus *Fusarium*. Several endophytic fungi also colonize wheat spikes asymptomatically. Pathogenic and commensal fungi share and compete for the same niche and thereby influence plant performance. Understanding the natural dynamics of the fungal community and how the pre-established species react to pathogen attack can provide useful information on the disease biology and the potential use of some of these endophytic organisms in disease control strategies. Fungal community composition was assessed during anthesis as well as during FHB attack in wheat spikes during 2016 and 2017 in two locations. Community metabarcoding revealed that endophyte communities are dominated by basidiomycete yeasts before anthesis and shift towards a more opportunistic ascomycete-rich community during kernel development. These dynamics are interrupted when *Fusarium* spp. colonize wheat spikes. The *Fusarium* pathogens appear to exclude other fungi from floral tissues as they are associated with a reduction in community diversity, especially in the kernel which they colonize rapidly. Similarly, the presence of several endophytes was negatively correlated with *Fusarium* spp. and linked with spikes that stayed healthy despite exposure to the pathogen. These endophytes belonged to the genera *Cladosporium*, *Itersonillia* and *Holtermanniella.* These findings support the hypothesis that some naturally occurring endophytes could outcompete or prevent FHB and represent a source of potential biological control agents in wheat.

**Electronic supplementary material:**

The online version of this article (10.1007/s00248-019-01426-3) contains supplementary material, which is available to authorized users.

## Introduction

Wheat (*Triticum aestivum*) is one of the most important crops in the world. Global production reaches around 750 million metric tons annually and it is a key cereal crop for human food supply [1]. Wheat productivity is vulnerable to diseases caused by fungal pathogens [2]. Fusarium head blight (FHB) is an economically important disease in wheat and other cereal crops [3]. It is caused by several species in the genus *Fusarium*, among which *F. graminearum*, *F. culmorum*, *F. poae*, *F. avenaceum*, *F. langsethiae* and *F. sporotrichioides* are generally considered the most important in Europe [4, 5]. FHB causes substantial yield losses by infecting and colonizing the spikes and the kernel during anthesis. The pathogens cause bleached lesions on the glumes and rachis and damage the grain [6]. More importantly, they produce specialized metabolites including trichothecenes and zearalenone (mycotoxins) [7]. These molecules facilitate infection and have been shown to play a role in competition with other microorganisms [8], but also reduce grain quality for consumption as they can cause health problems to humans and animals [9].

However, pathogens are not the only microorganisms colonizing plant tissues. Wheat is colonized by a highly diverse array of microorganisms including other fungal species, some of which live asymptomatically during their life within the plant [10]. These communities of endophytic organisms (endophytes) appear to be ubiquitous among species in the kingdom Plantae and are thought to interact closely with the plant as commensals or symbionts [11]. The importance of these communities in plant fitness has emerged in recent years [12] and certain of these fungi or whole communities in concert are considered to be involved in increased tolerance to stress [13, 14] and competition with pathogens [15].

Since these microorganisms possess similar characteristics and share essentially the same biological niche as pathogens, it is relevant to understand their relationships and behaviour during disease outbreaks [16]. Moreover, some of these microorganisms may have the potential to be exploited in disease control as biological alternatives or to complement current control options [17]. If fungal endophytes were to be exploited as a new source of biocontrol agents, it would necessitate a thorough understanding of their behaviour when the pathogen arrives and the disease occurs [18].

The main objective of this study was to assess the overall composition of the fungal endophytic community inside wheat spikes at the flowering stage and during FHB attack. Additionally, we aimed to detect endophytic species that could be associated to naturally occurring biological control. To our knowledge, this is the first report determining the endophytic fungal microbiome of different tissues during wheat spike development as well as the first analysis of the composition of these communities under FHB attack. These results reveal the magnitude of the pathogen-associated changes on the plant microbiome and support the concept that endophytic fungi may represent a new source of biocontrol agents against plant diseases.

## Materials and Methods

### Field Conditions

Trials were located at Flakkebjerg, Denmark (Aarhus University field station), 55°19′31.6′N 11°23′01.7′E and at Svalöv, Sweden (Lantmännen SW Seed AB), 55°54′39.8′N 13°07′09.3′E. In both locations, multiple winter wheat cultivars were tested for their response to FHB, under optimal infection conditions, for two consecutive years (2016 and 2017). Cultivars were grown in randomized 1 m^2^ plots and each cultivar had four replications. A diagram of the trial layout is shown in Supplementary Fig. 1.

### *Fusarium* spp. Inoculation

Trials were artificially infected with a mixture of *Fusarium* spp. (mainly *F. graminearum* and *F. culmorum* isolates from the area). A list of the species and isolates used at each location can be seen in Supplementary Table 1. In Denmark, spikes were sprayed directly with a *Fusarium* spp. macroconidial suspension (2 × 10^6^ spores/ml, 5 ml/m^2^) three times during anthesis (BBCH 60, 65 and 69, [19]) and irrigation was provided before and after inoculation. In Sweden, *Fusarium* spp. isolates were cultured on sterilized wheat and oat grains (imbibed for 16 h before inoculation) for 36 days. Kernels were then dried on paper towels for 4 days in a greenhouse. Once dried, the kernels were spread between plants before the flowering stage (BBCH 60) at a dose of 30 g/m^2^ and irrigation was not provided.

### Cultivar Selection and Sampling

FHB severity was assessed at 14 days after inoculation (BBCH 85). A visual linear scale from 0 to 10 was used to assess the percentage of infected plants within each plot (each level representing 10% increase in disease severity). A value was given to each plot and the mean of the four replications was calculated to determine infection severity for each cultivar. One cultivar was selected from each location during the first year, and the same cultivar was used during the second year. Cultivars KW-Nils and SW 14308 were selected for sampling in Denmark and Sweden, respectively, due to their low infection levels based on the visual assessment during the first year (5 and 13%, respectively). Severity for these two cultivars during the second year was 42% for KWS-Nils (Denmark) and 38% for SW14308 (Sweden). Spike sampling was performed at BBCH 90. In each of the four plots, four spikes with FHB symptoms (‘FHB-symptoms’) and four spikes with no symptoms (‘Symptomless’) were collected in order to obtain 16 spikes in for each *Fusarium*-status (FHB-symptoms or Symptomless). Spikes were selected throughout the plots avoiding the margins.

Additionally, 16 healthy spikes from the same cultivars growing in a non-inoculated adjacent plot were collected as a non-inoculated control (*Fusarium*-status: ‘Control’), see Supplementary Fig. 1. These plants were sown on the same date and were subjected to the same agricultural practices as the inoculated plots. Sampling in these plots was performed in a randomized way avoiding the margins.

During the second year, these adjacent, non-inoculated plots were also sampled at heading stage (BBCH 59) in order to assess the changes before and after anthesis in both studied location-cultivars combinations (Denmark-KWS-Nils and Sweden-SW-14308). Sixteen spikes were collected using the same sampling methodology. These spikes were designed as ‘Pre-anthesis’ samples. No Fusarium infection was observed in the adjacent plots during sampling.

### Sample Preparation

From each spike, five spikelets were removed from the middle section of the rachis and dissected. Glumes, lemmas and paleas were separated. Subsequently, these external bract tissues were pooled and designated as ‘Bracts’ independently from the kernels, designated as ‘Kernel’ in the analysis. Both types of tissues were surfaced sterilized using 96% ethanol for 1 min, 2% sodium hypochlorite for 3 min and 96% ethanol for 30 s followed by rinsing twice with sterile Milli-Q water. A volume of 100 μl from the last rinsing water was plated on potato dextrose agar plates (Difco™) to assess the efficiency of the surface sterilization. Tissue types (‘Bracts’, ‘Kernels’) from three spikes were pooled as one replication. Four replications for each *Fusarium*-status (FHB-symptoms, Symptomless, Control), tissue type (Bracts, Kernels) and location-cultivar (Sweden: cv. KWS-Nils, Denmark: cv. SW14308) were prepared for the first year (samples first year 48). During the second year, five replications were prepared for each group plus an additional condition: ‘Pre-anthesis’ samples (samples second year 80). Increment in the number of replications from year 1 to year 2 was due to availability of a fifth plot for the cultivars of interest during the second year (extra plot was sown for the cultivars selected during the first year). In total, 128 samples were prepared for downstream analysis.

### DNA Extraction and Library Preparation

Samples were freeze dried for 48 h and homogenized using ceramic beads (10 mm) in a mechanical shaker (SO-40a, Fluid Management Europe B.V.) using a 30-s programme three times. Approximately 100 mg of homogenized tissue per sample were used for DNA extraction using the E.Z.N.A.® Plant DNA Kit Plant (Omega Bio-tek, Norcross, GA, USA), following the manufacturer’s instructions. All the DNA samples were used for fungal library preparation. The fungal ITS2 region of the ribosomal RNA gene was amplified using the primers fITS7 and ITS4 [20]. Dual indexing was carried out to allow pooling of 96 samples. In addition, internal barcodes, consisting of varying numbers of nucleotides, were added to the forward primer for combining samples within each index combination [21, 22]. Primer sequences with internal barcodes and the index combinations are shown in Supplementary Table 2.

Each sample was amplified three times using different internal barcodes in order to ensure higher numbers of reads per sample. PCR reactions and pooling was carried as described previously [23]. In brief, PCR was performed in a reaction mixture of 25 μl consisting of the following: 5 μl PCR buffer Promega 5X (Promega Corporation, Madison, USA), 1.5 μl MgCl_2_ (25 mM), 2 μl dNTPs (2.5 mM each), 2 μl of each primer (10 μM), 0.125 μl (5 U/μl) GoTaq® Flexi polymerase (Promega Corporation, Madison, USA), 1 μl DNA template and 11.375 μl ddH_2_O. The PCR programme for the first reaction was 94 °C for 5 min followed by 30 cycles (94 °C for 30 s, 57 °C for 30 s, 72 °C for 30 s) and a final elongation step at 72 °C for 10 min. The PCR programme for the second reaction was 94 °C for 5 min followed by 10 cycles (94 °C for 30 s, 55 °C for 30 s, 72 °C for 30 s) and a final elongation step at 72 °C for 10 min. PCR products were pooled, ethanol precipitated and re-eluted in 50 μl Tris-EDTA buffer (10 mM, pH 8). The pooled DNA was stained with SYBR® Green I (Sigma-Aldrich, St. Louis, USA), separated on a 1.5% agarose gel and a band of the expected size (300–450 bp) was extracted using the QIAquick® Gel Extraction Kit (Qiagen, Hilden, Germany). The concentration was measured using a NanoDrop 2000 (Thermo Scientific™) and determined to be higher than 100 ng/μl. Sequencing was carried out at Eurofins MWG (Ebersberg, Germany) using the dual indexing Illumina Miseq platform. Raw read files from this study were deposited in the NCBI sequence read archive under the SRA accession: SRP184488.

### Bioinformatics and Statistical Analysis

Analysis of sequence reads was performed using QIIME version 1.9 [24]. The paired end reads were joined using SeqPrep, a fasq-join tool [25] with an overlapping minimum read length of 30 base pairs. The samples were split based on indexes and pooled to a single FASTA file, while removing reads with quality Phred scores less than 20 and other default parameters using the split_libraries_fastq.py command. Chimera detection, de-replication and clustering were done using ‘vsearch’ version 2.6 [26]. After excluding singletons, sequences were clustered to OTUs using a 97% similarity threshold (≥ 97%). Vsearch default options were used. Vsearch uses a distance-based greedy clustering method for clustering by default. Forward and reverse primers, internal barcodes and reads with less than 200 bp were removed. Supplementary Table 3 includes a detailed description of the number of reads in each step of the bioinformatics process. Taxonomy assignment for the clustered operational taxonomic units (OTUs) was done using the UNITE database version 7.2 [27]. In addition, the most abundant OTUs with at least 1000 reads were also blasted against the NCBI database and taxa assigned based on top 10 hits.

All statistical analyses were carried out in R version 3.5.2 [28]. Diversity-based analysis was carried out using the ‘vegan’ [29] and ‘phyloseq’ [30] packages available in R. The OTU table was either transformed to relative abundance or rarefied before performing alpha and beta diversity based calculations. For beta diversity and partitioning of variance, Bray-Curtis dissimilarity for fungal communities was subjected to a permutational multivariate analysis of variance (PERMANOVA) using the ‘adonis’ test from the ‘vegan’ package. Alpha diversity comparisons were made using a Kruskal-Wallis one-way analysis of variance and pairwise comparisons were made using a Wilcoxon signed-rank test with false discovery rate as a *P* value adjustment method.

Indicator species test was carried out using the Dufrene-Legendre Indicator Species Analysis in the ‘labdsv’ package [31]. Top 3 highest indicator value species under each condition were selected for Table 3. For co-occurrence networks, Spearman rank correlations among each OTU were carried out. Correlations with Spearman’s rho > 0.5 and < − 0.5 with *P* < 0.05 were considered as significantly correlated. All correlations were visualized using networks and network properties computed using the ‘igraph’ package [32]. All significantly correlated OTUs were used as nodes and the correlations values were used as edges.

## Results

Metabarcoding of the ITS2 region using DNA from wheat spike samples from both locations for both years resulted in a total of 8,216,141 reads. These reads clustered into 416 OTUs with 97% similarity after exclusion of non-fungal reads, using the UNITE database. A rarefaction curve indicated that sequencing coverage was successful in estimating most of the diversity in the communities (Supplementary Fig. 2). Across all samples, species from phylum Ascomycota represented 76.8% of the totally detected sequences and species from Basidiomycota 23.1%. The first 69 OTUs, representing 99.5% of the total diversity, were, where possible, identified to species level using the closest BLAST hit from NCBI database. Identities, GenBank accessions, coverage and percent of identity are shown in Supplementary Table 4. The most abundant species were *Fusarium graminearum* (OTU_1) and *Cladosporium herbarum* (OTU_2), which together represented approximately 60% of all the reads. Other abundant species were *Sporobolomyces roseus* (7.2%), *Vishniacozyma victoriae* (6.9%), *Fusarium culmorum* (4.9%), *Alternaria infectoria* (3.9%), *Cryptococcus tephrensis* (3%), *Aureobasidium pullulans* (1.4%) and *Parastagonospora nodorum* (1.4%).

### Natural Community Changes During Anthesis

**Fig. 1:**
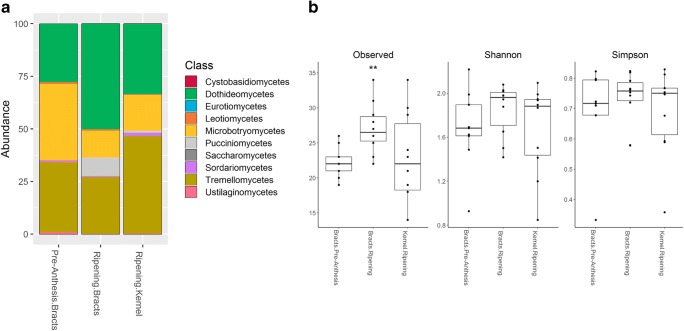
Endophytic fungal community composition in wheat external bracts (‘Bracts’) and ‘Kernels’ before and after anthesis. **a** Relative abundance of the top 10 most abundant fungal classes in wheat heads at ‘Pre-anthesis’ and ‘Ripening’. The reduction in relative abundance of Microbotryomycetes endophytic yeasts (yellow) at the ‘Ripening’ stage spikes is associated with an increase in Dothideomycetes (filamentous ascomycetes) (green) in the external bracts and a similar increase in Tremellomycetes (basidiomycetes) in the kernels (ochre). **b** Alpha diversity (Observed, Shannon and Simpson) changes across the flowering stage and different tissues. ‘Pre-anthesis’ bracts showed significantly less Observed alpha diversity than ‘Ripening’ bracts (**P < 0.01) using Wilcoxon signed-rank test

Fungal communities before and after anthesis comprised mainly genera such as *Cladosporium*, *Alternaria*, *Vishniacozyma* and *Sporobolomyces.* Community changes in relative abundance between flowering time and tissue type were observed at class level. The relative abundance of basidiomycete endophytic yeasts belonging to the class Microbotryomycetes decreased after anthesis in both ‘Bracts’ and ‘Kernels’ from nearly 40% to less than 20%. Inversely, Tremellomycetes yeast increased in relative abundance in the ‘Ripening’ kernels from 30% to almost 50%. Interestingly, ‘Ripening’ bracts showed a big increase in abundance of ascomycete Dothideomycetes, from nearly 25 to 50% (Fig. 1a). Community changes during flowering appeared to be associated with changes in abundance of pre-established fungal classes rather than changes in species identities.

Using three alpha diversity indices, we observed a slight increase in diversity from ‘Pre-anthesis’ spikes to ‘Ripening’ spikes. Indeed, ‘Bracts’ at ‘Ripening’ showed significantly higher Observed alpha diversity than ‘Bracts’ at ‘Pre-anthesis’ (*P* < 0.05) (Fig. 1b). Likewise, we observed that ‘Kernels’ harbour slightly less diversity than the ‘Bracts’, although no significant differences were detected. Interestingly, it was observed that spikes collected in Sweden (cv. SW 14308) showed higher diversity than those from Denmark (cv. KW-Nils) (*P* < 0.05) (Supplementary Fig. 3).

The fungal community structure from spikes before and after anthesis was compared using beta diversity values. PERMANOVA test showed that ‘Flowering stage’ (Pre-anthesis, Ripening) had a minor, yet significant effect on the community (*R*^2^ = 0.04, *P* < 0.01). Additionally, ‘Tissue type’ (Bracts, Kernel) was highly significant (*R*^2^ = 0.07, *P* < 0.01) just like ‘Location-Cultivar’ (Sweden: SW14308, Denmark: KWS-Nils) (*R*^2^ = 0.08, *P* < 0.01). A significant interaction between ‘Flowering stage’ and ‘Location-cultivar’ was also detected (*R*^2^ = 0.08, *P* < 0.01) (Table 1). Overall, the effect of ‘Flowering stage’ was small and exhibited a higher diversity in the ‘Bracts’ at the ‘Ripening’ stage.

**Table 1:** PERMANOVA test results on beta diversity for fungal community changes during anthesis

Factor	*R*^2^	*P* value
Flowering stage^a^	0.046	0.007
Tissue type^b^	0.073	< 0.01
Location-cultivar^c^	0.089	< 0.0001
Flowering stage × location-cultivar	0.085	< 0.0001

Effect of ‘Location’ and ‘Cultivar’ cannot be distinguished

^a^Flowering stage (Pre-Anthesis, Ripening)

^b^Tissue type (Bracts, Kernels)

^c^Location-cultivar (Sweden: cv. SW14038, Denmark: cv. KWS-Nils)

### FHB-Induced Community Changes

**Fig. 2:**
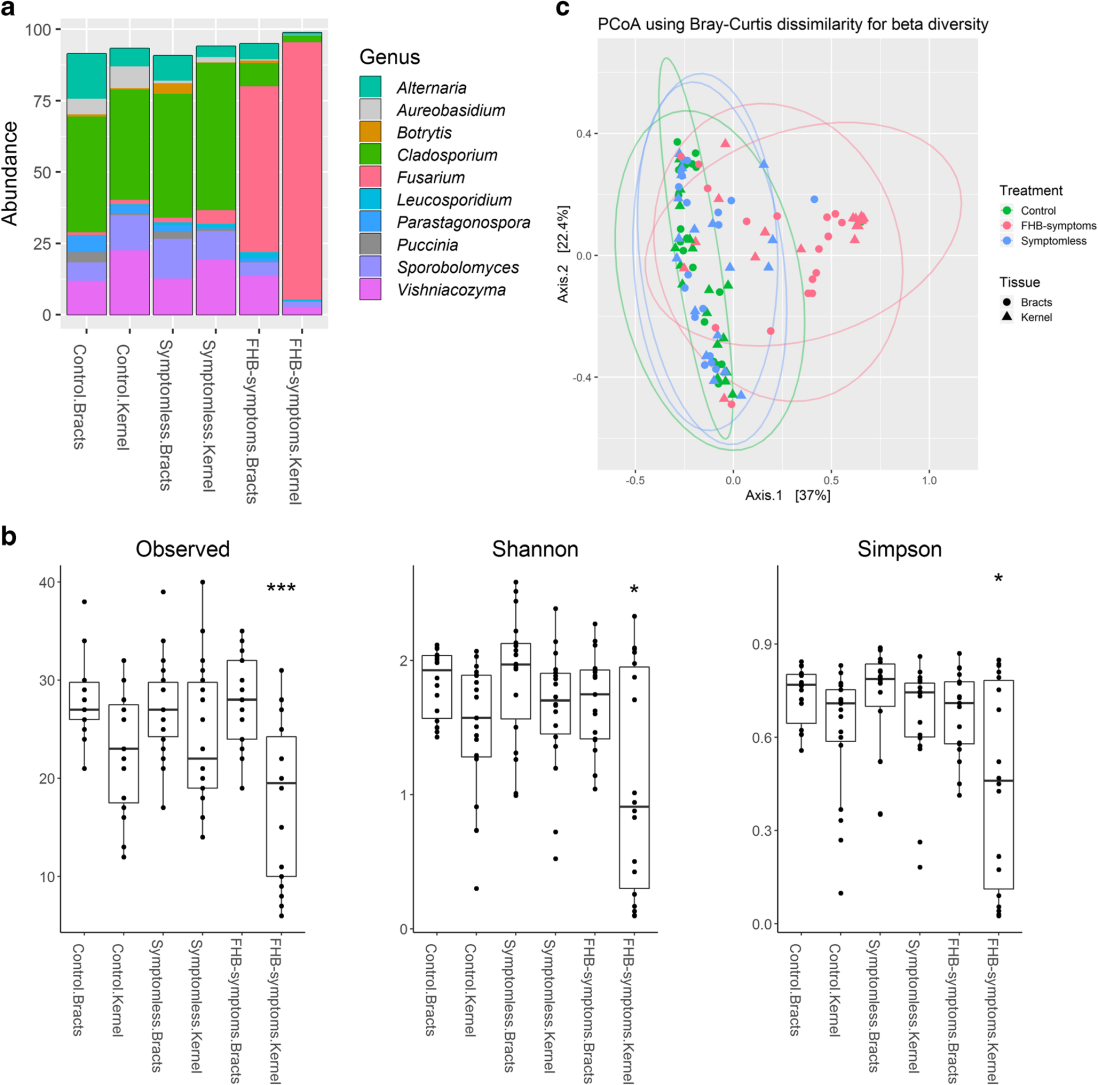
Endophytic fungal community composition in wheat external bracts (‘Bracts’) and ‘Kernels’ during FHB attack. **a** Relative abundance of the top 10 most abundant fungal genera in wheat spikes from different groups: ‘FHB-symptoms’, ‘Symptomless’ and ‘Control’ (not inoculated). *Fusarium* infection is associated with changes in the community structure in ‘FHB-symptoms’ spikes and was correlated to reduced abundance of other fungi, especially in ‘Kernels’. *Cladosporium* and *Vishniacozyma* were the most abundant genera in ‘Control’ and ‘Symptomless’ spikes. **b** Alpha diversity (Observed, Shannon, Simpson) for each condition and tissue. ‘FHB-symptoms’ kernels showed less diversity (*P < 0.05, ***P < 0.001) using Kruskal-Wallis test. **c** PCoA using Bray-Curtis dissimilarity for beta diversity during FHB attack. ‘FHB-symptoms’ spikes clustered independently from other samples. Ellipses represent 95% confidence intervals

Fungal communities in ‘Control’ and ‘Symptomless’ spikes showed a similarly diverse community composition. Relative abundance at the genus level showed that filamentous ascomycetes like *Cladosporium*, *Parastagonospora* and *Alternaria* (Dothideomycetes) represented more than 50% of the fungal abundance. However, *Cladosporium* was slightly more abundant in the ‘Symptomless’ bracts and kernels. On the other hand, endophytic basidiomycete yeasts (*Vishniacozyma* and *Sporobolomyces*, Tremellomycetes) were also abundant, but present in considerably lower proportions (near 15%) in the samples with no visible FHB infection (‘Control’ and ‘Symptomless’). This diversity contrasts with the one observed in ‘FHB-symptoms’ spikes, where *Fusarium* spp. represented up to 90% of the total fungal abundance. Interestingly, *Fusarium* spp. were also present, but at a lower proportion in the ‘Symptomless’ spikes, where they did not exceed 5% of the total relative abundance (Fig. 2a).

Increased relative abundance of *Fusarium* was associated with a reduced abundance of other ascomycete fungi such as *Cladosporium*, *Alternaria* as well as some basidiomycetes like *Sporobolomyces* and *Vishniacozyma*. This tendency was similar for both tissue types ‘Kernels’ and ‘Bracts’, but especially clear in the kernels where *Fusarium* abundance reached almost 95%. Genera such as *Alternaria*, *Botrytis* and *Parastagonospora* were more abundant in the external bracts than in the kernel. On the contrary, genera such as *Fusarium, Cladosporium* and *Vishniacozyma* were more abundant in the kernels compared to the external bracts. Additionally, a small increase in the abundance of *Botrytis* was observed in ‘FHB-symptoms’ and ‘Symptomless’ spikes compared to the ‘Control’. Overall, *Fusarium* infection was associated to a reduced abundance of most fungi inside wheat spikes.

Alpha diversity indices showed that ‘FHB-symptoms’ spikes had slightly less diversity than ‘Symptomless’ and ‘Control’ spikes. When looking at the interaction between ‘*Fusarium-*status’ and ‘Tissue type’, we observed that ‘FHB symptoms’ kernels showed lower alpha diversity than ‘Control’ and ‘Symptomless’ tissues (*P* < 0.05) (Fig. 2b). Similarly, we observed that in ‘FHB-symptoms’ spikes, fungal diversity was significantly lower during 2017 than in 2016 (*P* < 0.05) (Supplementary Fig. 3).

A PERMANOVA analysis showed that ‘*Fusarium*-status’ (FHB-symptoms, Symptomless or Control) accounted for most of the variation (12%), with a high significance (*R*^2^ = 0.12, *P* < 0.001). Similarly, ‘Tissue type’ (Bracts, Kernel) with 3% (*R*^2^ = 0.03, *P* < 0.001), ‘Year’ (2016, 2017) with 6% (*R*^2^ = 0.06, *P* < 0.001) and ‘Location-Cultivar’ (Sweden: SW14308, Denmark: KWS-Nils) had a considerable effect (*R*^2^ = 0.01, *P* < 0.001). Multiple interactions between factors were also detected in the community structure (Table 2). A principal coordinates analysis, based on Bray-Curtis dissimilarity of beta diversity, showed that ‘FHB-symptoms’ and ‘Year’ were the main two main drivers of the variation in the samples (Fig. 2c). In general, FHB symptoms were associated with a reduction in fungal species diversity in the spikes, especially inside infected kernels.

**Table 2:** PERMANOVA test results on beta diversity for fungal community changes during FHB

Factor	*R*^2^	*P* value
*Fusarium*-status^a^	0.119	< 0.0001
Tissue^b^	0.030	< 0.0001
Year^c^	0.068	< 0.0001
Location-cultivar^d^	0.019	< 0.0001
*Fusarium*-status × year	0.082	< 0.0001
*Fusarium*-status × location	0.0046	< 0.0001

Effect of ‘Location’ and ‘Cultivar’ cannot be distinguished

^a^*Fusarium*-status (FHB-symptoms, Symptomless, Control)

^b^Tissue type (Bracts, Kernels)

^c^Year (2016, 2017)

^d^Location-cultivar (Sweden: cv. SW14038, Denmark: cv. KWS-Nils)

### Indicator Species of Different Communities

**Fig. 3:**
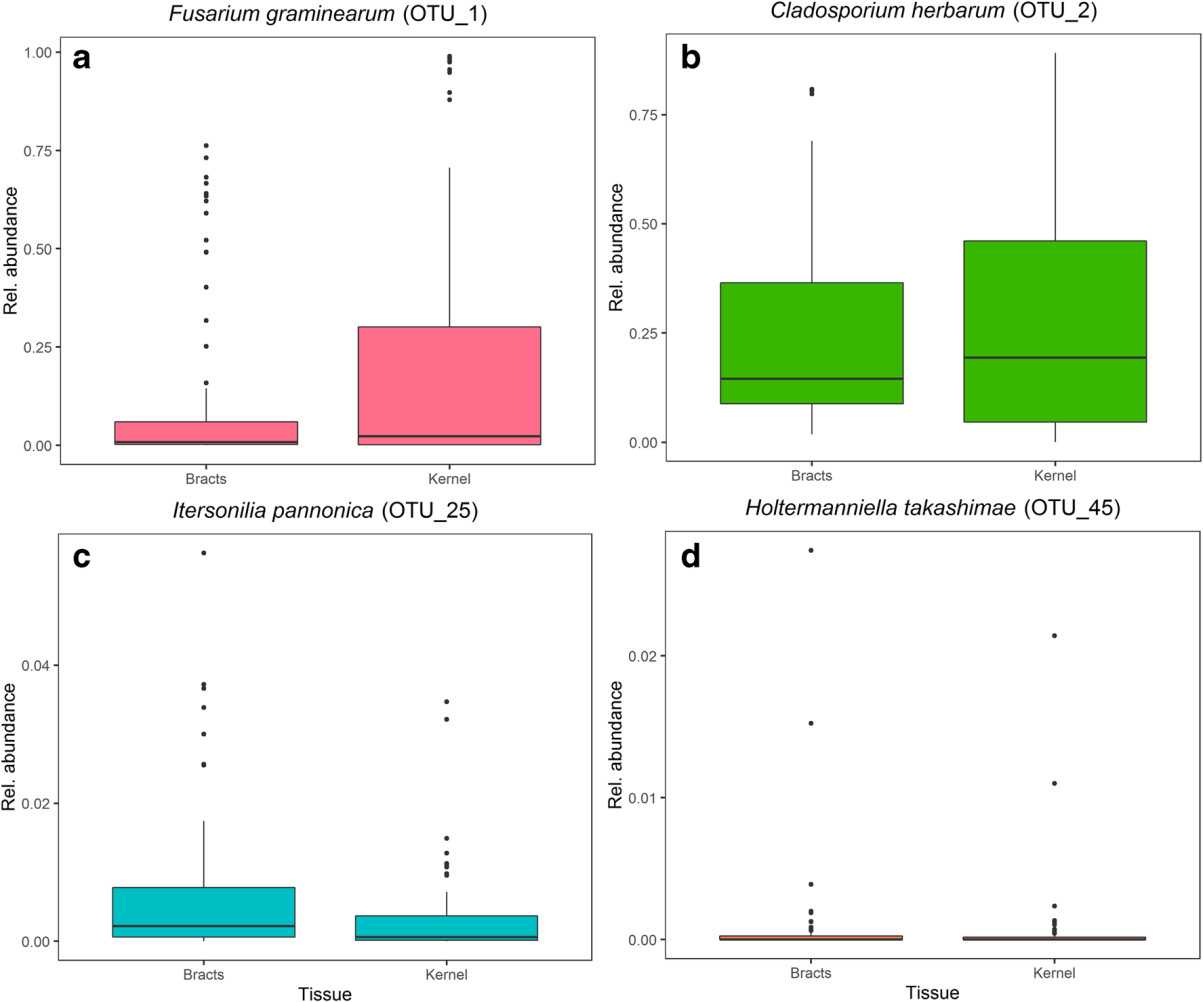
Relative abundance of four different fungal OTUs inside wheat spike tissues. **a***Fusarium graminearum* (OTU_1) was detected in higher abundance in kernels. **b***Cladosporium herbarum* (OTU_2) was observed in higher proportion in kernels than in the external bracts. **c***Itersonilia pannonica* (OTU_25) was more abundant in the ‘Bracts’ than in the ‘Kernels’. **d***Holtermanniela takashimae* (OTU_45) showed low abundance both in ‘Kernels’ and ‘Bracts’. Only *C. herbarum* appear to compete for the same biological niche as the pathogens

An indicator species test was performed comparing *Fusarium*-status communities (FHB-symptoms, Symptomless and Control) to determine key species in each group. The top 3 OTUs in each comparison of indicator species test are presented in Table 3. As expected, *Fusarium* spp. were the dominant species in the ‘FHB-symptoms’ spikes. At least 13 different OTUs belonging to the genus *Fusarium* were strong indicators of the spikes with symptoms when compared to the ‘Control’ and the ‘Symptomless’ spikes. Similarly, when assessing the indicator species of the ‘Control’ spikes against ‘FHB-symptoms’, only *Cladosporium herbarum* (OTU_2) emerged as an indicator of ‘Control’ spikes. Moreover, we observed that *C. herbarum* (OTU_2) was present in both ‘Bracts’ and ‘Kernels’, but it was more abundant in the ‘Kernels’ similar to *Fusarium graminearum* (OTU_1) (Fig. 3). Interestingly, within the ‘Symptomless’ spikes, different OTUs such as *Itersonilia pannonica* and *Holtermanniella takashimae* were strong indicators compared to ‘FHB-symptoms’. *Holtermanniella takashimae* was also a strong indicator species when comparing ‘Symptomless’ and ‘Control’ spikes. These two OTUs were less abundant and more frequently found in the external bracts.

**Table 3:** Dufrene-Legendre Indicator Species test from each of the Fusarium status population comparisons

Comparison	*Fusarium*-status^a^	OTU	Indicator value	Closest BLAST hit
‘FHB-Symptoms’	‘FHB-Symptoms’	OTU_1	0.87	*Fusarium graminearum*
- ‘Symptomless’		OTU_396	0.69	*Fusarium culmorum*
		OTU_494	0.63	*Fusarium culmorum*
	‘Symptomless’	OTU_2	0.72	*Cladosporium herbarum*
		OTU_377	0.30	*Holtermanniella takashimae*
		OTU_79	0.25	*Itersonilia pannonica*
FHB-Symptoms	‘FHB-Symptoms’	OTU_399	0.92	*Fusarium culmorum*
- Control		OTU_1	0.91	*Fusarium graminearum*
		OTU_396	0.79	*Fusarium culmorum*
	Control	OTU_2	0.71	*Cladosporium herbarum*
Symptomless	Symptomless	OTU_11	0.77	*Leucosporidium fragarium*
- Control		OTU_45	0.59	*Holtermanniella takashimae*
		OTU_377	0.30	*Holtermanniella takashimae*
	Control	No species detected		

^a^*Fusarium*-status (spikes with FHB-symptoms, Symptomless and Control)

### Fungal OTUs Interactions Inside Wheat Spikes

**Fig. 4:**
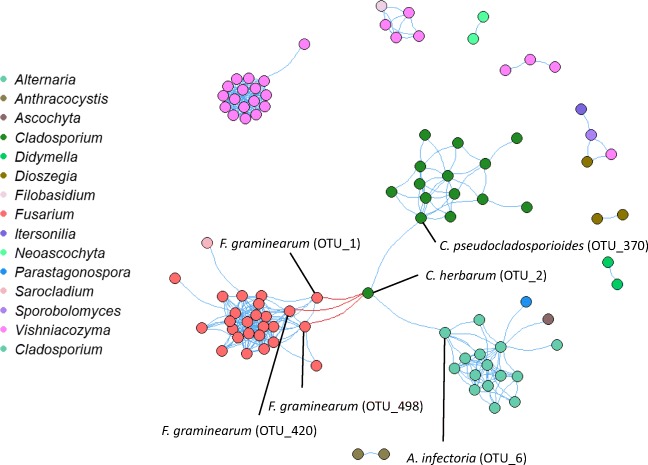
Network plot for co-occurrence between different OTUs identified in wheat spikes in this study. Positive correlations are shown as blue edges and negative correlations as red edges between different OTUs (nods). OTUs from the same genera are visualized with the same colour. Four fungal genera interacted closely with each other: *Fusarium* (pink), *Alternaria* (light blue), *Vishniacozyma* (purple) and *Cladosporium* (dark green). *Cladosporium herbarum* (OTU_2) negatively correlated with three *Fusarium graminearum* OTUs

Co-occurrence analysis was used to detect specific interactions between OTUs in the communities. Most of the different OTUs positively correlated within their own genus. This was the case for the genera *Fusarium* (pink cluster), *Vishniacozyma* (purple cluster), *Cladosporium* (dark green cluster) and *Alternaria* (light blue cluster) (Fig. 4)*.* Positive correlations across genera were observed between *Cladosporium herbarum* (OTU_2) and *Alternaria infectoria* (OTU_6). Interestingly, *C. herbarum* (OTU_2) also negatively correlated with three *Fusarium graminearum* OTUs, including the most abundant, *F. graminearum* (OTU_1). These negative correlations were observed both in the ‘Kernels’ and in the ‘Bracts’. The correlations with their respective *P* values can be seen in Supplementary Table 5.

## Discussion

A plethora of fungal species live unseen within plants tissues. These organisms, either alone or as communities, are believed to play an important role in plant fitness and therefore in crop performance. Understanding the way these endophytic communities respond to disease could broaden our knowledge of plant pathogens and pave the way for potential new disease control strategies based on biological control. To the best of our knowledge, this study represents the first analysis of fungal communities in wheat spikes under Fusarium head blight attack. Moreover, this is the first report of endophytic communities in wheat since previous studies have not attempted to distinguish between epiphytes and endophytes in wheat tissues. Using sodium hypochlorite surface-sterilized tissues, we enriched the samples with endophytic fungi in the analysis by removing most of the external microbiota [33].

We observed how closely associated endophytic communities inside wheat spikes tend to increase in diversity as anthesis occurs and kernel develop. Additionally, we identified a succession from a basidiomycete yeast-dominated community before anthesis to a filamentous ascomycete-rich community during ripening. Most importantly, we showed how *Fusarium* infection interacts with these dynamics. The pathogens quickly colonize tissues and are associated with a reduction in fungal diversity, especially inside the newly formed kernels.

Fungal communities in wheat tissues (root, crowns, tillers, leaves, glumes, awns and anthers) have been assessed using culture-dependent methods in the past [9, 34, 35]. These approaches have only examined the limited cultivable communities selected by the isolation media. Most of these studies have identified between 30 and 50 fungal species and have shown minor representation of phylum Basidiomycota (1–2%) [34, 35]. On the other hand, most culture-independent studies, including this one, have detected higher numbers of species and have observed that basidiomycete diversity constitutes nearly 25% of the total fungal species [36–38], but this can change depending on the ITS amplification primers used [39]. In general, culture-dependent methods have been shown to isolate and identify the main players in the wheat mycobiome but the resolution to detect variations and specific interactions remains low. Culture-independent methods provide a better image of the communities and their dynamics.

Our data reveal that changes in fungal communities during flowering are quantitative rather than qualitative. We observed a reduction in abundance of Microbotryomycetes yeasts (*Sporobolomyces* and *Leucosporidium*) at the ripening stage. This reduction was associated with an increase in abundance of Tremellomycetes yeasts (*Vishniacozyma*, *Cryptococcus*, *Itersonilia*, *Dioszegia*) in the ‘Kernels’ and Dothideomycetes (largest group of ascomycota) in the ‘Bracts’.

Similar dynamics have also been observed in fungal communities on wheat leaves and stems (including epiphytes). Dothideomycete species increased in abundance while Microbotryomycetes and Tremellomycete species were reduced from pre-flowering (BBCH 45) to seed development (BBCH 85) [37, 38]. For wheat spikes, it has been observed that the ratio of ascomycetes to basidiomycetes increased during kernel development [36]. Dothideomycete species such as *Cladosporium cladiosporoides*, *Alternaria infectoria* and *Parastagonospora avenae* were the most abundant of these fungi in our study. These have been reported as pathogens in other cereals, but most commonly as later stage saprophytes on wheat. Other studies have demonstrated that wheat spikes and grains harbour a high diversity of these ascomycetes [36, 39, 40]. Similar trends have been observed in wheat leaves as *Zymoseptoria tritici* was found to be the most abundant fungus in later stage leaves [23, 41]. This suggests a general increase in abundance of opportunistic or even pathogenic fungi over time in wheat tissues. Our data indicate that this shift in fungal communities also occurs inside wheat spikes and that it commences during anthesis, earlier than previously reported. Interestingly, we showed that ascomycete enrichment was not detectable in wheat kernels. This suggests that fungal diversity in external tissues such as glumes and lemmas (‘Bracts’) increase over time as they are environmentally exposed to airborne spores while the kernels appear to be protected from these changes, at least during early ripening.

Using two methods of inoculation during the flowering stage, it was possible to measure the effect of FHB on fungal communities in wheat head tissues. The *Fusarium* isolates that were used for inoculation rapidly colonized the tissues and established in the community. The fungi caused visible disease at 14 days after inoculation and this was coupled to significant changes in the fungal community. Increasing abundance of *Fusarium* (above 80%) in the infected spikes was associated with a reduced abundance of other fungi, representing both ascomycetes and basidiomycetes. This process was similar for both ‘Kernels’ and ‘Bracts’, but was especially noticeable in the ‘Kernels’.

A few studies have assessed the effect of pathogens on established microbial communities in plant tissue. Evaluation of the fungal diversity of wheat leaves showing symptoms of *Stagonospora nodorum* blotch reported the co-existence of several pathogens in these symptomatic leaves, but there was no comparison against healthy leaves [42]. Recently, it was shown that the most abundant endophytic bacterial genera in *Fusarium*-infected wheat spikes increased in relative abundance (from 2 to 10 fold) compared to healthy spikes under field conditions [43]. Similarly, bacterial diversity increased in *Paullinia cupana* leaves affected by anthracnose symptoms (*Colletotrichum* spp.) compared to leaves with no symptoms [44]. A similar tendency was detected, although not statistically significant, for bacterial communities in tomato roots attacked by the nematode *Meloidogyne incognita* when compared to non-infected roots [45].

Opportunistic bacteria can benefit from pathogen attack, but in the case of fungi, our data support the hypothesis that the *Fusarium* spp. either prevent other fungi from establishing and growing through direct competition inside wheat spikes and/or they hinder the growth of the rest of the community by modifying the environment inside the tissue to their favour. The *Fusarium*-wheat molecular interaction has been described to occur in two phases: a first phase (asymptomatic) where the pathogen metabolism focuses on plant-defence suppression and growth and a second phase where nutrient depletion activates the production of cell-degrading enzymes [3]. Likewise, production of mycotoxins, specifically deoxynivalenol (DON), has been observed during the early stage [46]. Interestingly, it has been shown that *Fusarium* infection induces production of putrescine, a DON promoter, in the wheat spikes [47]. This suggests an active role of the pathogen in changing the internal chemical conditions of the spike during infection. Indeed, the known role of specialized metabolites, such as the mycotoxins DON and zearalenone, is consistent with their ability to outcompete other fungi [48, 49]. We observed that diversity was drastically reduced in the ‘Kernels’ while remaining similar or slightly higher in the ‘Bracts’ during symptomatic infection. Symptomless spikes (which showed low levels of *Fusarium* abundance) did not show such a reduction. This suggests that the reduction in endophyte diversity associated to *Fusarium* infection is likely to occur during the second phase of infection and is mediated by the physiological changes caused by the production of enzymes and mycotoxins in the kernel.

Finally, one of the objectives of this study was to identify potentially naturally occurring endophytic fungi associated with healthy spikes. We collected spikes that remained visually healthy after pathogen exposure (‘Symptomless’) and compared their endophytic communities to the ones in spikes with symptoms ‘FHB-symptoms’. A prospective candidate would theoretically be enriched in ‘Symptomless’ spikes and/or negatively correlated with *Fusarium* spp. We identified *Cladosporium herbarum*, *Holtermanniella takashimae* and *Itersonilia pannonica* as indicator species of the ‘Symptomless’ spikes. Additionally, we detected a strong negative correlation between *Cladosporium herbarum* and three *Fusarium graminearum* OTUs.

*Cladosporium herbarum* is a common endophyte and an environmentally ubiquitous saprophytic fungus [50]. It has been associated with cereals and isolated from wheat tissues in several studies [9] and has even been reported to be pathogenic in some wheat cultivars [51]. We found higher *C. herbarum* abundance in ‘Kernels’, similar to *Fusarium* spp. The observed negative correlation between these two species suggests that *C. herbarum* and *F. graminearum* compete for the same biological niche. It is worth noting that we found high *C. herbarum* abundance in spikes with no visible symptoms. Similar negative interactions with *Fusarium* spp. have been observed for other ascomycete fungi such as *Parastagonospora nodorum* in grains and tillers [38]. However, this was seen in a study where *Fusarium* infection was not recorded. Interestingly, several studies have tested *Cladosporium* spp. as biocontrol agents. *Cladosporium cladosporioides* and *C. pseudocladosporioides* were shown to have antagonistic effects against *Puccinia horiana*, a pathogen in chrysanthemum [52]. However, *Cladosporium halotolerans* has been tested against *F. graminearum* in wheat with little success [53].

On the other hand, *Holtermanniella takashimae*, *Itersonilia pannonica* and other tremellomycetous yeasts have been found to be ubiquitous colonizers of plant tissues [54]. These two species were detected in both the ‘Kernels’ and the ‘Bracts’, but they were most abundant in the ‘Bracts’. This means that they do not necessarily compete with *Fusarium* spp. for the same niche, but could rather prevent *Fusarium* infection as they appear to be enriched in healthy plants. Tremellomycete yeasts, including the genus *Cryptococcus*, have been shown to provide growth enhancement to plants [55]. In fact, the yeast isolate *Cryptococcus flavescens*, strain OH 182.9, isolated from wheat anthers, reduced *Fusarium* disease severity and mycotoxin accumulation in wheat and furthermore increased kernel weight compared to the infected control [56, 57]. This isolate was also observed to be able to colonize the abaxial surfaces of glume and lemma tissues [58], the same tissues where we observed them in higher abundance. Whether *C. herbarum*, *H. takashimae* or *I. pannonica* outcompete or prevent *F. graminearum* colonization of wheat spikes remains to be determined. However, our data suggest they could potentially be involved in a naturally occurring biocontrol effect against FHB.

The new microbiome perspective to plant-microbe interactions allows a more holistic approach to plant disease control and opens the door to unexplored ways of biological control. FHB results in an imbalance on the fungal communities in wheat spikes. Theoretically, it will be possible to modify the wheat microbiome with beneficial microorganisms that counterbalance the specialized mechanisms of pathogens to maintain system homeostasis. Future work must aim to identify more of these interactions in natural FHB-infested areas.

## Electronic Supplementary Material

ESM 1 (PDF 358 kb)

ESM 2 (XLSX 104 kb)
