# Supplementary material for: Fusarium Head Blight Modifies Fungal Endophytic Communities During Infection of Wheat Spikes
Source: Microb Ecol. 2019 Aug 26;79(2):397–408. doi: 10.1007/s00248-019-01426-3 (PMC7033075; doi:10.1007/s00248-019-01426-3)
Supplement: Supplementary file 1 — (PDF 358 kb) [file 248_2019_1426_MOESM1_ESM.pdf]

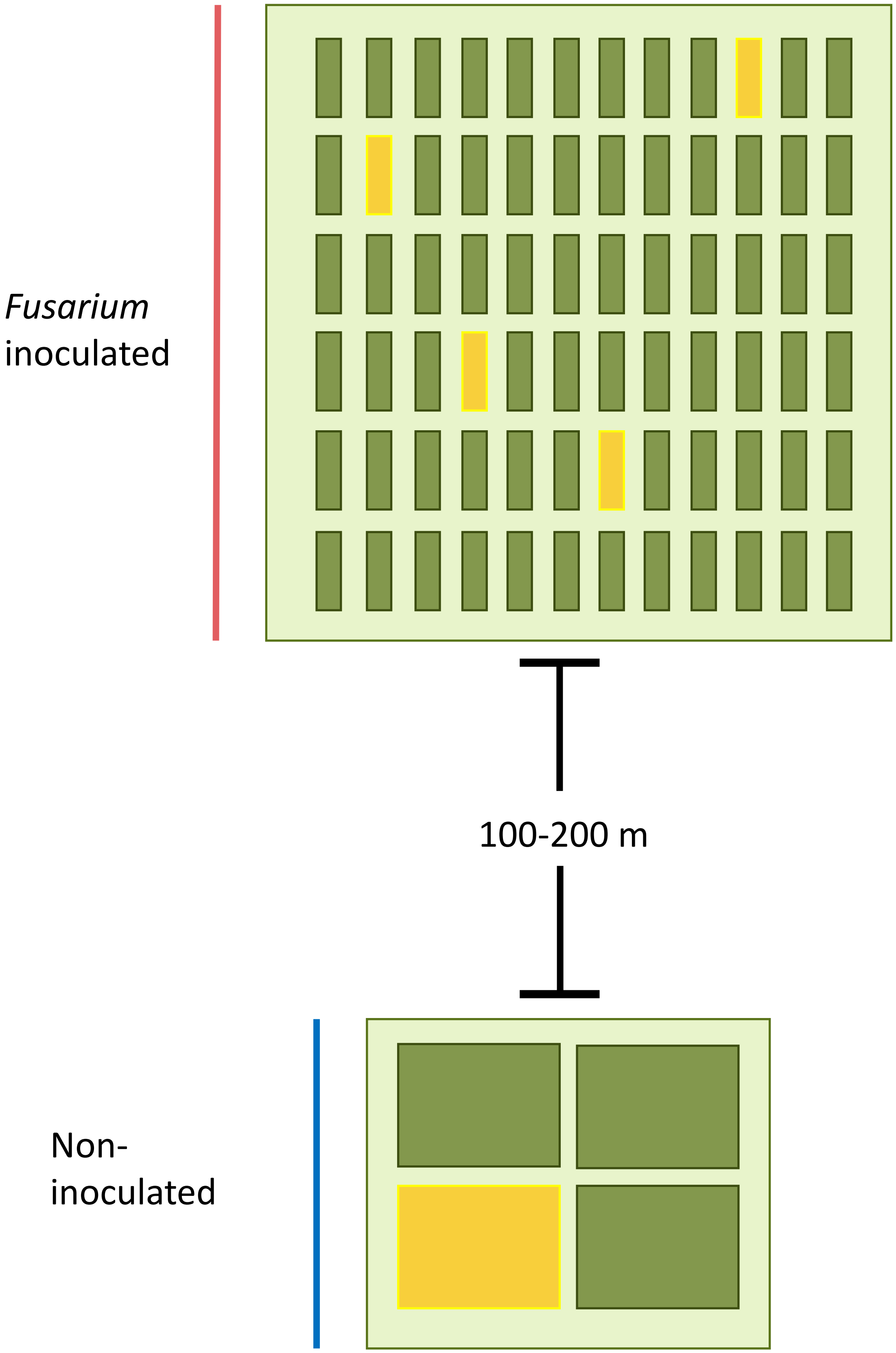

Supplementary Figure 1. Fusarium trials layout representation. Multiple cultivars are tested on a small area. Four replicates (1m^2) are randomly distributed within the plot. During sampling , one cultivar in each Location was selected (yellow). From each replicate, 4 spikes with symptoms (FHB-symptoms) and four symptomless (Symptomless) were harvested (16 in total). Sixteen non-inoculated spikes (Control) were collected from an adjacent plot where these same cultivars are grown. Plants from these plots were sown on the same date and received same agronomical practices. Same experimental methods were used in both locations during two years.

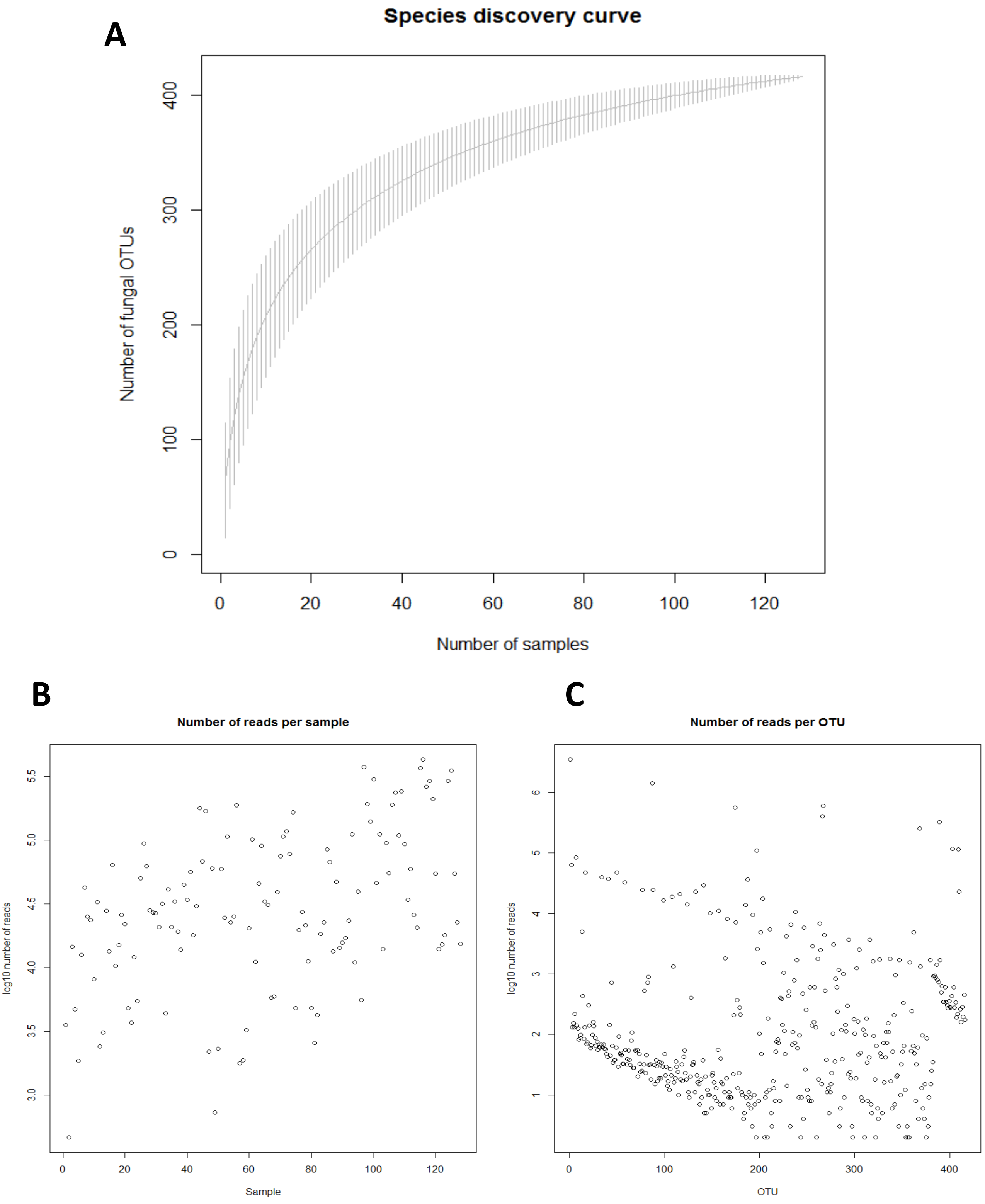

Supplementary Figure 2. A. Species recovery curve across samples. **B.** Number of reads per sample. **C.** Number of reads across OTUs

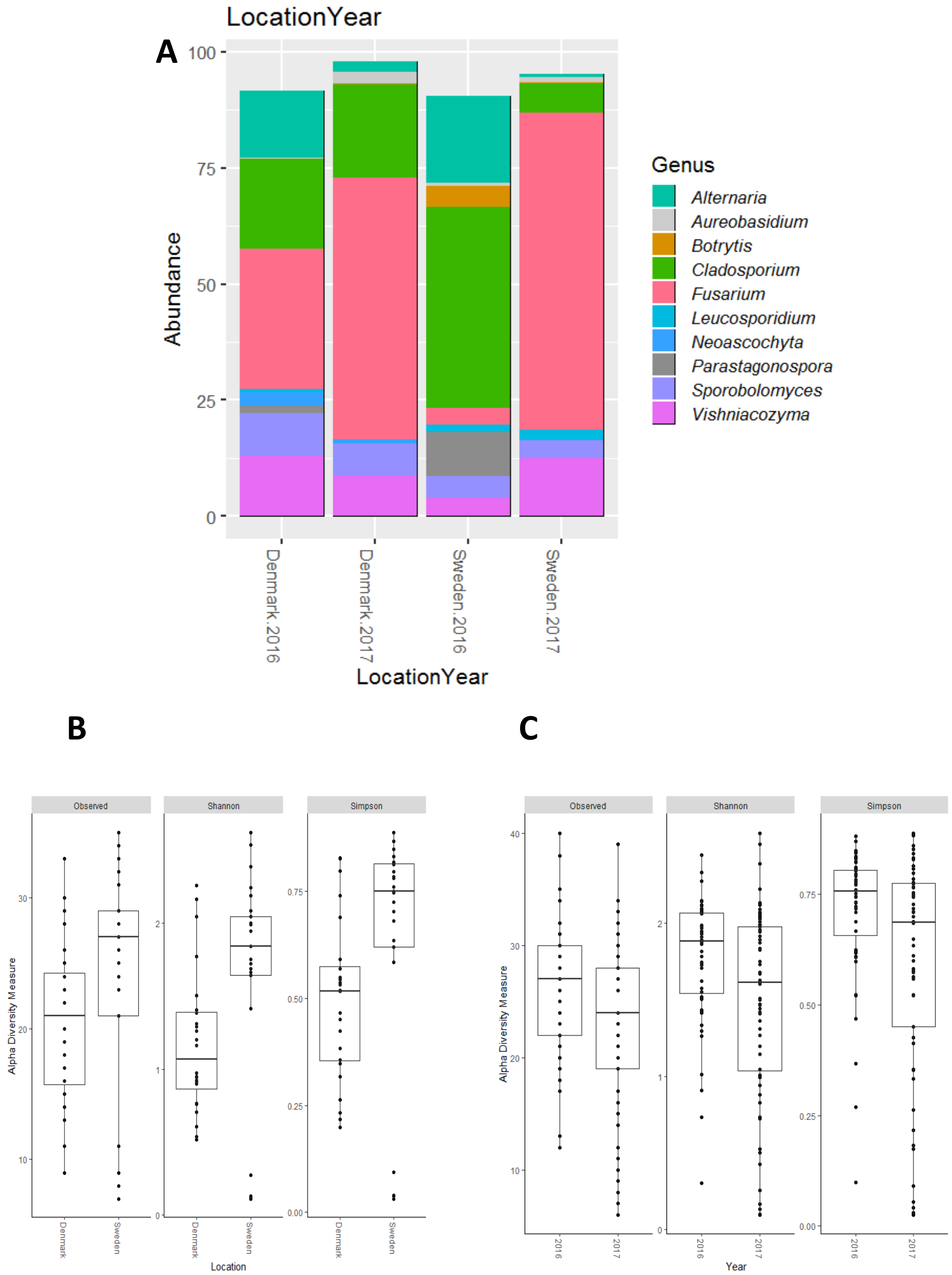

Supplementary Figure 3 **A.** Relative abundance of the top-ten most abundant genera for each location-year combination. **B.** Alpha diversity under three indices for each location. **C.** Alpha diversity under three indices for each year
